# Supratentorial infarcts accompanying hiccup

**DOI:** 10.1002/brb3.1439

**Published:** 2019-10-15

**Authors:** Ryo Itabashi, Kaoru Endo, Takuya Saito, Kazuki Fukuma, Yukako Yazawa

**Affiliations:** ^1^ Department of Stroke Neurology Kohnan Hospital Sendai Japan

**Keywords:** cerebrovascular diseases, hiccup, stroke

## Abstract

**Backgrounds:**

The main culprit lesion causing hiccup in patients with ischemic stroke is thought to involve the medulla oblongata, but some cases of hiccups caused by damage to the supratentorial cortex have been reported. The present study aimed to address the clinical and radiological characteristics of acute stroke patients accompanied by hiccups caused by supratentorial lesions.

**Method:**

We retrospectively studied 5,309 consecutive patients with acute ischemic stroke or transient ischemic attack who were admitted to our institute within 7 days after onset between April 2006 and September 2017. We searched for the term “hiccup” in prospectively collected descriptive datasets and analyzed associations between hiccup and clinical and radiological findings, with particular focus on patients with supratentorial lesions.

**Results:**

We finally selected 16 stroke patients accompanied by hiccup. Nine patients had infarcts in the lateral medulla oblongata, and others had supratentorial infarcts (three patients with cortical infarcts, four patients with subcortical infarcts). Moreover, the right hemisphere was frequently damaged in this series (6/7, 86%).

**Conclusions:**

Hiccup could be caused by supratentorial infarcts including the insular cortex, temporal lobe, and subcortex.

## Graphical abstract

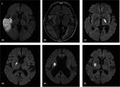

## 1 INTRODUCTION

The hiccup (singultus) is an involuntary spasmodic contraction of the diaphragm accompanied by sudden closure of the glottis, producing a familiar and peculiar “hic” sound (Launois, Bizec, Whitelaw, Cabane, & Derenne, 1993). The reflex arch for hiccup is thought to consist of an afferent pathway, the hiccup center, and an efferent pathway. The afferent pathway may be the phrenic nerve, the vagus nerve, or sympathetic afferents from T6 to T12, and the efferent pathway is primarily the phrenic nerve (Kahrilas & Shi, 1997; Launois et al., 1993; Marsot‐Dupuch, Bousson, Cabane, & Tubiana, 1995; Wagner & Stapczynski, 1982; Yamazaki, Sugiura, & Kurokawa, 2008). Although the neuroanatomical hiccup center has not been fully illuminated, the brainstem, probably through interactions with the respiratory center, phrenic nerve nuclei, medullary reticular formation, and hypothalamus, is postulated to be the hiccup center between the afferent and efferent pathways (Arita, Oshima, Kita, & Sakamoto, 1994). On the other hand, a nonspecific anatomic location in the spinal cord between C3 and C5 segments has also been postulated to play a role as a neuroanatomical center for hiccup (Kahrilas & Shi, 1997; Launois et al., 1993; Marsot‐Dupuch et al., 1995).

The hiccup would be caused by stimulation, probably in the form of injury or irritation, of one or more components in the hiccup system (Kahrilas & Shi, 1997; Launois et al., 1993; Marsot‐Dupuch et al., 1995; Wagner & Stapczynski, 1982; Yamazaki et al., 2008). The main causes of hiccup can be classified as: central nervous system; psychiatric; metabolic; toxic and infectious; ear, nose, and throat disease; thoracic; and abdominal (Kahrilas & Shi, 1997; Launois et al., 1993; Marsot‐Dupuch et al., 1995). Although the main culprit lesion in the central nervous system causing hiccup is thought to involve the brainstem, including the medulla oblongata or pons (al Deeb, Sharif, al Moutaery, & Biary, 1991; Kim, 2003; Kobayashi et al., 2009; Kumar & Dromerick, 1998; Liu, Fuh, & Wang, 2008; Mattana, Mattana, & Roxo, 2010; Musumeci, Cristofori, & Bricolo, 2000; Park et al., 2005), a few cases with persistent or intractable hiccups caused by damage to the supratentorial cortex have been reported (van Durme, Idema, & van Guldener, 2008; Jansen, Joosten, & Vingerhoets, 1990; Lee, Pritchard, & Weiner, 2011; Longatti, Basaldella, Moro, Ciccarino, & Franzini, 2010; Marsot‐Dupuch et al., 1995; Tiedt & Wenzel, 2013). However, the characteristics of patients showing hiccups due to supratentorial lesions have not yet been fully elucidated. The present study aimed to address the clinical and radiological characteristics of acute stroke patients presenting with hiccup at a single stroke center, with a particular focus on supratentorial lesions associated with hiccups.

## 2 METHODS

### 2.1 Study population

Subjects in this present study were selected from 5,309 consecutive patients with acute ischemic stroke or transient ischemic attack (TIA) who were admitted to Kohnan Hospital (Sendai, Miyagi, Japan) within 7 days after onset between April 2006 and September 2017. All patients admitted to our institute during this period were examined by neurologists, neurosurgeons, or both and were screened by routine laboratory tests, as well as computed tomography (CT) or magnetic resonance imaging (MRI). Based on the findings from clinical examinations and brain imaging, board‐certified stroke neurologists specializing in the care of stroke patients made a diagnosis of ischemic stroke or TIA. The severity of neurological deficits was evaluated using the National Institutes of Health Stroke Scale (NIHSS) score on admission (Lyden et al., 1994).

Clinical and investigative data were prospectively entered in a standardized fashion by stroke neurologists into the Kohnan Hospital Stroke Registry. Among the collected data in the registry, history of illness, neurological findings, and clinical course during hospital stay were recorded descriptively and used as components for the discharge summaries. These descriptive records were retrospectively searched by a board‐certified consultant stroke neurologist (R. I.) regarding history of illness, neurological findings, and clinical course during the hospital stay for the term “hiccup” (the actual search was for the Japanese equivalent to hiccup, “Shakkuri” or “Kitsugyaku,” with the former as the colloquial term, and the latter as the medical term) in October 2017. We did not limit the duration of hiccup for inclusion in this study. The medical records of these screened patients were reviewed by a single reviewer (R. I.) to exclude patients who had other possible causes, including metabolic, abdominal, or thoracic disorders. The Kohnan Hospital Ethics Committee approved the study protocol. Due to the retrospective nature of the study, the need for written informed consent was waived.

### 2.2 Analysis

We analyzed the association between hiccup and clinical characteristics, including age, sex, accompanying neurological findings, and radiological findings from brain imaging. Because of the small number of patients initially screened in this analysis, we did not adopt statistical analyses to compare characteristics between patients with and without hiccup. As a particular focus was placed on patients with supratentorial lesions, characteristics including radiological findings were described for each case.

## 3 RESULTS

**Figure 1:**
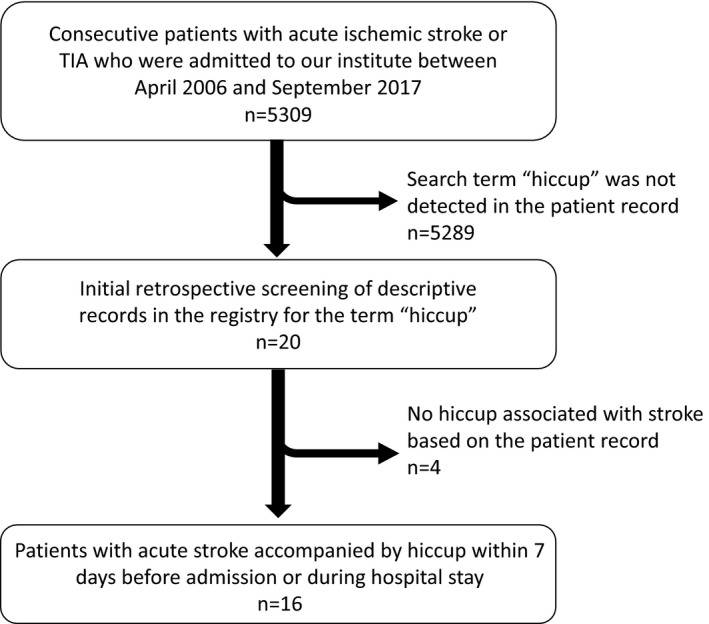
Flow chart for patient selection

**Figure 2:**
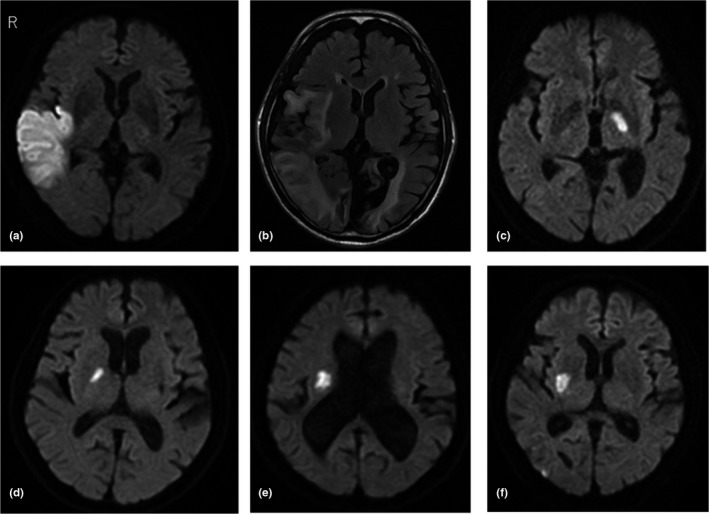
Radiological findings for patients with supratentorial infarcts. We could not obtain radiological data for Patient 1. (a) Diffusion‐weighted imaging (DWI) for Patient 2, obtained 4 days after onset. (b) Fluid‐attenuated inversion recovery imaging in Patient 3, obtained 6 days after onset. (c) DWI in Patient 4, obtained 3 days after onset. (d) DWI in Patient 5, obtained 3 days after onset. (e) DWI in Patient 6, obtained 4 days after onset. (f) DWI in Patient 7, obtained 1 day after onset

After searching the registry, 20 patients with acute stroke were initially screened. Just after initial screening, two cases had been excluded because the extracted description from the database indicates that there was no hiccup in the cases. Although we reviewed the medical records of the screened patients, there were no patients with hiccup due to other possible causes, such as metabolic, abdominal, or thoracic disorders. One case was excluded because of suspicion whether it was mix‐up the hiccup with respiratory distress symptom. Another case was excluded because we could not confirm the episode of hiccup in the medical records. We finally selected 16 stroke patients (median age, 64.5 years; male 88%) accompanied by hiccup within 7 days before admission or during the hospital stay (Figure 1). Median initial NIHSS score was 3 (interquartile range [IQR], 1–8.25). Median interval from stroke onset to occurrence of hiccup was 3.5 days (IQR, 0–6 days), and median duration of hiccup was 4 days (IQR, 2.25–10 days). Among these 16 patients, nine patients showed infarcts in the lateral medulla oblongata, and the remaining seven patients had supratentorial infarcts. Clinical and radiological characteristics of these seven patients with supratentorial infarcts are shown in Table 1 and Figure 2. Three patients had cortical infarcts including the right insular cortex and temporal lobe (Table 1, Figure 2a,b). On the other hand, four patients had subcortical infarcts in the anterior circulation. The posterior limb of the inner capsule, basal ganglia, and corona radiata were injured in these patients (Table 1, Figure 2c‐f). Although Patient 4 (Figure 2c) showed cerebellar infarct contralateral to the inner capsule infarct, the lesion in the cerebellum was small, and the brainstem was unaffected. Notably, the right side was damaged in six of the seven patients with supratentorial injury (86%).

**Table 1:** Clinical findings of patients with supratentorial infarcts

No.	Age	Sex	Initial NIHSS	Duration of hiccup	Other neurological signs	Side	Infarct site
1	64	M	18	10 days	Disorientation USN CD Hemiparesis SD	Right	Whole territory in the MCA
2	76	M	6	4 days	Disorientation AD Anosognosia DA USN Hemiparesis SD	Right	Insular cortex STG MTG
3	85	M	12	3 days	Somnolence USN CD Hemiparesis SD	Right	Insular cortex IFG MTG IOG
4	65	M	2	4 days	Aphasia Hemianopsia	Left (IC) Right (CB)	Posterior limb of IC CB
5	71	M	0	2 days	Hemiparesis	Right	GP
6	80	M	9	4 days	Disorientation Extinction Hemiparesis SD	Right	CR
7	71	M	11	25 days	Dysarthria USN CD Dysphagia Hemiparesis	Right	Putamen CR IOG

Abbreviations: AD, attention disorder; CB, cerebellum; CD, conjugate deviation; CR, corona radiata; DA, dressing apraxia; GP, globus pallidus; IC, inner capsule; IFG, inferior frontal gyrus; IOG, inferior occipital gyrus; M, male; MCA, middle cerebral artery; MTG, middle temporal gyrus; NIHSS, National Institutes of Health Stroke Scale; SD, sensory disturbance; STG, superior temporal gyrus; USN, unilateral spatial neglect.

## 4 DISCUSSION

We demonstrated that the culprit lesion in some patients with hiccup caused by acute ischemic stroke was partially attributable to supratentorial infarcts. We identified not only cortical infarcts including the insular cortex and temporal lobe, as previously reported to be associated with hiccup (van Durme et al., 2008; Jansen et al., 1990; Lee et al., 2011; Longatti et al., 2010; Marsot‐Dupuch et al., 1995; Tiedt & Wenzel, 2013), but also supratentorial subcortical infarcts. Moreover, the right hemisphere was frequently damaged in this series.

Hiccup is one of the common symptoms in patients with lateral medullary infarction. Kim reported that one of 4 patients among a large series with lateral medullary infarction showed hiccup (Kim, 2003). On the other hand, a few cases with supratentorial cortical damage including the temporal lobe or insular cortex have been reported to experience intractable hiccups (van Durme et al., 2008; Jansen et al., 1990; Lee et al., 2011; Longatti et al., 2010; Marsot‐Dupuch et al., 1995; Tiedt & Wenzel, 2013). We described four cases with hiccup caused by supratentorial subcortical infarcts, in addition to three cases with cortical infarcts that were in accordance with past studies. Interestingly, subcortical infarcts were located close to the insular cortex in two cases with hiccup (Patient 6, Figure 2e; Patient 7, Figure 2f).

Subcortical regions adjacent to the insular cortex including the basal ganglia, inner capsule, and corona radiata might be involved in inhibitory control of the reflex arch of the hiccup system. Damage to the central nervous system is thought to cause hiccup by releasing the higher‐center inhibition of the hiccup reflex (Kahrilas & Shi, 1997; Launois et al., 1993; Marsot‐Dupuch et al., 1995; Wagner & Stapczynski, 1982; Yamazaki et al., 2008). Discontinuation of inhibitory control by cortical regions would affect the brainstem involved in hiccup generation, resulting in altered sympathetic tone (Tiedt & Wenzel, 2013). On the other hand, some brain regions including the insula, brainstem, and supratentorial subcortex adjacent to the insular cortex were reported to be associated with Takotsubo cardiomyopathy, which is related to failure of the autonomic control of cardiac activity (Yoshimura et al., 2008). Moreover, the right insula was implicated in the autonomic control of cardiac activity (Colivicchi, Bassi, Santini, & Caltagirone, 2004). In the literature, there were five cases with the right‐sided, three with the left‐sided, and one case with diffuse bilateral lesions among nine cases with hiccup associated the supratentorial injury (van Durme et al., 2008; Jansen et al., 1990; Lee et al., 2011; Longatti et al., 2010; Marsot‐Dupuch et al., 1995; Tiedt & Wenzel, 2013). The predominance of the right‐sided infarct in our study could be attributable to a chance finding; however, the importance of the right hemisphere about the pathogenesis of hiccup in stroke could not be ignored. A cortical visceral network comprising the insular cortex and temporomesial structures was advocated based on a study with electrocortical stimulation of the anterior insular cortex (Ostrowsky et al., 2000). Injuries to the insular cortex or adjacent subcortex in the pathogenesis of hiccup in stroke could be attributable to the same mechanisms as cases with cardiac autonomic failure in stroke.

This study had some limitations. The single‐center design was one. Despite the information from the large database, we could not obtain an enough number of patients with hiccup to perform statistical analysis to address the detailed role of supratentorial lesions for hiccup. Although data from the Kohnan Stroke Registry were collected in a standardized, preplanned fashion, evaluation of hiccup was not performed in a systematic way because of its retrospective nature. Therefore, it could not be proven that the hiccup associated with stroke was comprehensively picked up from the population. Because mild or short‐term hiccup in patients with brainstem lesion could be considered as common and of no importance symptom, it might have been overlooked. Moreover, this study collected cases with hiccup regardless of duration, whereas hiccup cases in the literature almost always involved persistent or intractable symptoms. Therefore, it is possible that the association between the supratentorial lesion and the hiccup had been overestimated. Nonetheless, the present study was the first to evaluate the significance of supratentorial subcortical infarct in association with hiccup in patients with acute stroke. To address more detailed and robust association between infarct location and hiccup pathophysiology, a large, prospective registry focusing on hiccup is warranted.

## CONFLICT OF INTEREST

None declared.

## AUTHOR CONTRIBUTIONS

Ryo Itabashi involved in data acquisition, analyzed the data, and wrote the manuscript. Kaoru Endo, Takuya Saito, Kazuki Fukuma, and Yukako Yazawa involved in data acquisition and critical revision of the manuscript.

## Data Availability

The data that support the findings of this study are available on request from the corresponding author. The data are not publicly available due to ethical restrictions.

## References

[brb31439-bib-0001] al Deeb, S. M. , Sharif, H. , al Moutaery, K. , & Biary, N. (1991). Intractable hiccup induced by brainstem lesion. Journal of the Neurological Sciences, 103, 144–150. 10.1016/0022-510X(91)90157-3 1880531

[brb31439-bib-0002] Arita, H. , Oshima, T. , Kita, I. , & Sakamoto, M. (1994). Generation of hiccup by electrical stimulation in medulla of cats. Neuroscience Letters, 175, 67–70. 10.1016/0304-3940(94)91079-0 7970214

[brb31439-bib-0003] Colivicchi, F. , Bassi, A. , Santini, M. , & Caltagirone, C. (2004). Cardiac autonomic derangement and arrhythmias in right‐sided stroke with insular involvement. Stroke, 35, 2094–2098. 10.1161/01.STR.0000138452.81003.4c 15272134

[brb31439-bib-0004] Jansen, P. H. , Joosten, E. M. , & Vingerhoets, H. M. (1990). Persistent periodic hiccups following brain abscess: A case report. Journal of Neurology, Neurosurgery and Psychiatry, 53, 83–84. 10.1136/jnnp.53.1.83 PMC1014105 2303837

[brb31439-bib-0005] Kahrilas, P. J. , & Shi, G. (1997). Why do we hiccup? Gut, 41, 712–713. 10.1136/gut.41.5.712 9414986 PMC1891574

[brb31439-bib-0006] Kim, J. S. (2003). Pure lateral medullary infarction: Clinical‐radiological correlation of 130 acute, consecutive patients. Brain, 126, 1864–1872. 10.1093/brain/awg169 12805095

[brb31439-bib-0007] Kobayashi, Z. , Tsuchiya, K. , Uchihara, T. , Nakamura, A. , Haga, C. , Yokota, O. , … Mizusawa, H. (2009). Intractable hiccup caused by medulla oblongata lesions: A study of an autopsy patient with possible neuromyelitis optica. Journal of the Neurological Sciences, 285, 241–245. 10.1016/j.jns.2009.06.014 19577262

[brb31439-bib-0008] Kumar, A. , & Dromerick, A. W. (1998). Intractable hiccups during stroke rehabilitation. Archives of Physical Medicine and Rehabilitation, 79, 697–699. 10.1016/S0003-9993(98)90047-8 9630152

[brb31439-bib-0009] Launois, S. , Bizec, J. L. , Whitelaw, W. A. , Cabane, J. , & Derenne, J. P. (1993). Hiccup in adults: An overview. European Respiratory Journal, 6, 563–575. 8491309

[brb31439-bib-0010] Lee, M. H. , Pritchard, J. M. , & Weiner, W. J. (2011). Clinical reasoning: A 44‐year‐old man with a 3‐month history of hiccups. Neurology, 77, e145–e148. 10.1212/WNL.0b013e31823d7652 22170949

[brb31439-bib-0011] Liu, F. C. , Fuh, J. L. , & Wang, S. J. (2008). Symptomatic trigeminal autonomic cephalalgia associated with allodynia in a patient with multiple sclerosis. Journal of the Chinese Medical Association, 71, 583–586. 10.1016/S1726-4901(08)70174-6 19015058

[brb31439-bib-0012] Longatti, P. , Basaldella, L. , Moro, M. , Ciccarino, P. , & Franzini, A. (2010). Refractory central supratentorial hiccup partially relieved with vagus nerve stimulation. Journal of Neurology, Neurosurgery and Psychiatry, 81, 821–822. 10.1136/jnnp.2009.179929 20581144

[brb31439-bib-0013] Lyden, P. , Brott, T. , Tilley, B. , Welch, K. M. , Mascha, E. J. , Levine, S. , … Marler, J. (1994). Improved reliability of the NIH Stroke Scale using video training. NINDS TPA stroke study group. Stroke, 25, 2220–2226. 10.1161/01.STR.25.11.2220 7974549

[brb31439-bib-0014] Marsot‐Dupuch, K. , Bousson, V. , Cabane, J. , & Tubiana, J. M. (1995). Intractable hiccups: The role of cerebral MR in cases without systemic cause. AJNR. American Journal of Neuroradiology, 16, 2093–2100. 8585500 PMC8337219

[brb31439-bib-0015] Mattana, M. , Mattana, P. R. , & Roxo, M. R. (2010). Intractable hiccup induced by cavernous angioma in the medulla oblongata: Case report. Journal of Neurology, Neurosurgery and Psychiatry, 81, 353–354. 10.1136/jnnp.2009.175273 20185478

[brb31439-bib-0016] Musumeci, A. , Cristofori, L. , & Bricolo, A. (2000). Persistent hiccup as presenting symptom in medulla oblongata cavernoma: A case report and review of the literature. Clinical Neurology and Neurosurgery, 102, 13–17. 10.1016/S0303-8467(99)00058-X 10717396

[brb31439-bib-0017] Ostrowsky, K. , Isnard, J. , Ryvlin, P. , Guénot, M. , Fischer, C. , & Mauguière, F. (2000). Functional mapping of the insular cortex: Clinical implication in temporal lobe epilepsy. Epilepsia, 41, 681–686. 10.1111/j.1528-1157.2000.tb00228.x 10840399

[brb31439-bib-0018] Park, M. H. , Kim, B. J. , Koh, S. B. , Park, M. K. , Park, K. W. , & Lee, D. H. (2005). Lesional location of lateral medullary infarction presenting hiccups (singultus). Journal of Neurology, Neurosurgery and Psychiatry, 76, 95–98. 10.1136/jnnp.2004.039362 PMC1739304 15608002

[brb31439-bib-0019] Tiedt, H. O. , & Wenzel, R. (2013). Persistent hiccups as sole manifestation of right cortical infarction without apparent brainstem lesion. Journal of Neurology, 260, 1913–1914. 10.1007/s00415-013-6960-9 23681648

[brb31439-bib-0020] van Durme, C. M. , Idema, R. N. , & van Guldener, C. (2008). Two rare complications of glioblastoma multiforme: Persistent hiccup and acquired haemophilia A. Netherlands Journal of Medicine, 66, 286–288. 18663257

[brb31439-bib-0021] Wagner, M. S. , & Stapczynski, J. S. (1982). Persistent hiccups. Annals of Emergency Medicine, 11, 24–26. 10.1016/S0196-0644(82)80009-7 7055350

[brb31439-bib-0022] Yamazaki, Y. , Sugiura, T. , & Kurokawa, K. (2008). Sinister hiccups. The Lancet, 371, 1550 10.1016/S0140-6736(08)60660-1 18456105

[brb31439-bib-0023] Yoshimura, S. , Toyoda, K. , Ohara, T. , Nagasawa, H. , Ohtani, N. , Kuwashiro, T. , … Minematsu, K. (2008). Takotsubo cardiomyopathy in acute ischemic stroke. Annals of Neurology, 64, 547–554. 10.1002/ana.21459 18688801

